# Evaluation of a new lymphocyte proliferation assay based on cyclic voltammetry; an alternative method

**DOI:** 10.1038/s41598-019-41171-8

**Published:** 2019-03-14

**Authors:** Mohammad Nikbakht, Babak Pakbin, Gholamreza Nikbakht Brujeni

**Affiliations:** 1 Department of Electronic Engineering, School of Electrical and Computer Engineering, University of Tehran, Tehran, Iran; 2 Department of Microbiology and Immunology, Faculty of Veterinary Medicine, University of Tehran, Tehran, Iran

## Abstract

Lymphocyte proliferation assays are widely used to assess the cell-mediated immunity. Current *in vitro* testing methods that are being used have extensive applications but still more problematic, due to the technical complexity and the needs for specialized equipment and reagents. Electrochemical methods such as cyclic voltammetry represent a very promising tool for the development of label-free *in vitro* assays of cell proliferation and viability. Here, a novel procedure based on voltammetric behaviours of proliferating cells was fabricated. Results indicated that proliferation in cell cultures and whole blood can be monitored electrochemically using cyclic voltammetry. In the comparison with colorimetric (MTT) assay, cyclic voltammetry gave the best correlation with cell count data over a range of 1200–300,000 cells/well of a microplate. Besides the advantages of short assay duration (4 hours) and the rapidness, the possibility use of fresh blood without further processing, would give more accurate results because cells are monitoring in an intact environment. Cyclic voltammetry assay is an efficient analytical method, which can provide a simple platform for the electrochemical study of lymphocyte proliferation.

## Introduction

Humoral and cell-mediated immunity (CMI) are two major components of the innate and adoptive immune responses. Humoral immune response involves the synthesis and release of macromolecules, such as antibodies, cytokines, antimicrobial peptides, complement and acute phase proteins into the blood and other body fluids. In contrast, CMI is mediated by activated or sensitized reticuloendothelial (Phagocytic) and lymphoid (Lymphocytes) cells^1^. Most immunological studies necessitate evaluation of both humoral and CMI responses. Humoral components can be easily monitored by use of precipitation, agglutination, neutralization and enzyme-linked immunosorbent assay (ELISA) methods. Assessment of CMI can be undertaken by *in vivo*, *in situ* and *in vitro* methods. Delayed-type hypersensitivity skin testing is an example of *in vivo* assessment of cellular immunity. Cells of immune system can also be evaluated and enumerated *in situ* by immunofluorescence microscopy, immunohistochemistry, or flow cytometry. Methods for *in vitro* evaluating the CMI are broadly grouped into the stimulation (e.g. leucocyte migration technique), proliferation, cytotoxicity and effector activity (cytokine expression) assays.

The proliferation and/or cytotoxic assays is widely used for potency determination of vaccines, diagnosis of infectious disease^2–5^, immune deficiencies and drug discoveries^4,6,7^. In practical terms, cells are stimulating by mitogen, antigen, toxin or drug, which may cause the cell activation or death. Then measurement of proliferating and/or surviving cells is conventionally determined by radiometric or colorimetric (Dye based) assays^8^. These procedures have widespread potential applications but still more problematic, due to the technical complexity and the needs for specialized equipment and reagents.

Electrochemical methods such as cyclic voltammetry (CV) represent a very promising tool for the development of label-free *in vitro* assays for evaluation of cell proliferation and viability^9,10^. Electrochemical sensors operate by reacting with the analyte of interest to produce an electrical signal proportional to the analyte concentrations. A typical electrochemical sensor consists of a sensing electrode (working electrode) and a reference electrode separated by an electrolyte. For most applications, a three-electrode system is used with the reference connected to a high-input-impedance given a constant voltage and a counter electrode is to measure the current flow. The cyclic voltammetry, as a type of potentiodynamic electrochemical measurement, is used for analytes that are redox active within the potential window to be scanned. It is a proficient tool that screen the intrinsic redox reaction of the electrode material as the potential of the electrode is swept in a cyclic manner. Particularly, redox reactions catalyzed by proteins or enzymes to maintain energy and major natural mechanisms. The living cell has its own redox properties that undergo alterations of metabolisms, products and membrane events during the activation and replication or apoptosis (cell death). Therefore, it can be speculated that proliferation of lymphocytes leads to change in their redox properties which might be monitored by electrochemical or conductometer cell sensor. It has been demonstrated that redox properties in living cells can be determined by CV and the peak current increased by increase of cell number^11^. Using CV for proliferation assays will also provide information on the thermodynamics of redox processes and electron-transfer reactions. Cyclic voltammetry waves virtually fingerprint the individual electrochemical properties of redox systems^12,13^.

At the present study, we describe cyclic voltammetry measurement of human lymphocyte proliferation, as an alternative means of colorimetric lymphocyte proliferation assay.

## Results

**Figure 1:**
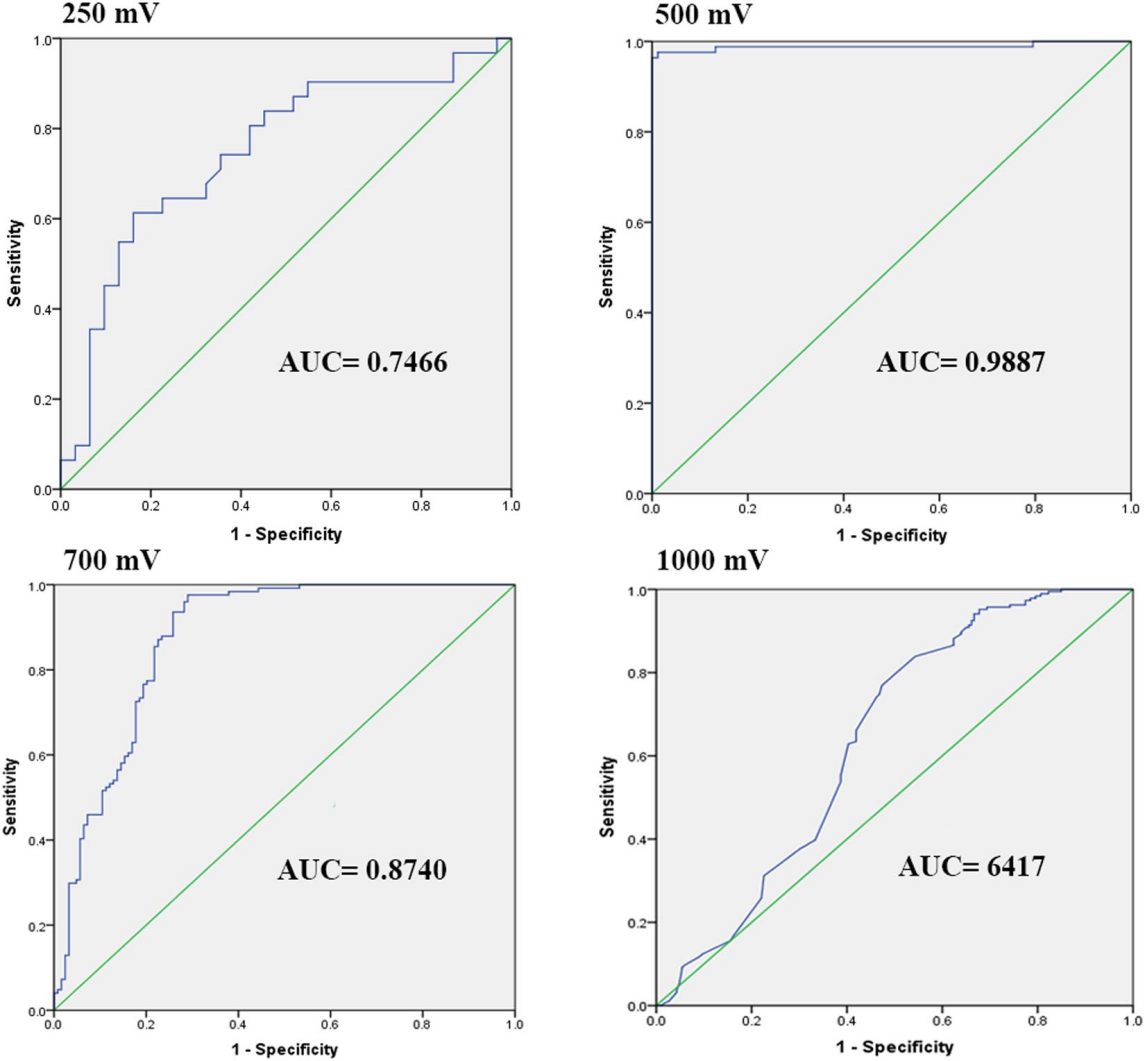
Determining a cut-off value for assigning lymphocyte proliferation responses. Graph represents ROC curves analysis of the results obtained from cyclic voltammetry assay. Four different potential sets including 250, 500, 750 and 1000 mV, at 5 mV/sample, 100 mV/sec slope were tested. In each assay at least 40 test and 40 control well were tested. AUC, area under the ROC curve.

Cyclic voltammetry was used to characterize the cell proliferation and to determine the cell concentration. To evaluate the optimum test parameters and CV index cutoff, we used ROC curves approach for comparing different potential sets (i.e. 250, 500, 750 and 1000 mV) (Fig. 1), and to find a criterion for determining mitogen-induced responses in the cell proliferation assay (Table 1). The area under the ROC curve (AUC) represents the test accuracy and is equal to the probability of correctly discriminating between affected and non-affected cells. The best AUC (0.9887) obtained for the electrochemical responses between 0 and 500 mV, at 5 mV/sample sweep rate and 100 mV/sec slope.

**Table 1:** Area under the ROC curve (AUC values), sensitivity, specificity and Youden’s index of the different potentials calculated for deduced cut-off values.

Electrode potential	AUC	Cut-off (mV)	Sensitivity range (%)	Specificity range (%)	*J*
0–250	0.7466	116–250	61.3–74.2	61.3–70.6	0.35–0.42
0–500	0.9887	348–454	91–6–97.6	97.6–100	0.91–0.95
0–750	0.8740	280–575	93.5–97.6	70.2–74.2	0.65–0.68
0–1000	0.6417	183–410	74.2–90.9	34.9–53.8	0.26–0.28

**Figure 2:**
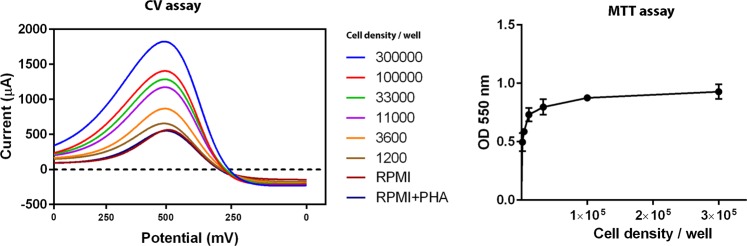
Differential voltammograms and MTT OD values for different concentrations of PBMC ranging from 3 × 10^6^ to 1.2 × 10^4^ cells/mL (3 × 10^5^ to 1.2 × 10^3^ cells/well). Complete RPMI (RPMI-1640 medium supplemented with 10% FBS and 1% penicillin/streptomycin) used to dilute the cells. Complete RPMI was used as the blank and an equal amount of PHA (5 µg/mL) were added to the RPMI in wells corresponding to PHA + RPMI.

When the electrode potential was set to 0 and 500 mV (AUC = 0.99%), the best pairs of values for highest sensitivity and specificity was found at several current values (Table 1; Fig. 2). Our results indicated that the current values obtained at the range of 348–454 mV (23 spot) appear to have the best discriminating power to differentiate mitogen-induced cells from control subjects. To enable comparison between treated and control cells in proliferation assays the formula 1 was used to calculate CV index:

1 $$CVI({\mu }_{CV})=\frac{{\sum }^{}\frac{{{\rm{c}}}_{is}}{{{\rm{c}}}_{rpmi}}}{N}$$

Where µ is a general mean; c_is_ the current of the CV ith spot in the sample (test or control); c_rpmi_ the current of the CV ith spot in the RPMI media; N the number of spots. Finally, the stimulation index for CV method (CVSI) is expressed with average CVI value in the mitogen induced group divided by average CVI value in negative controls.

**Figure 3:**
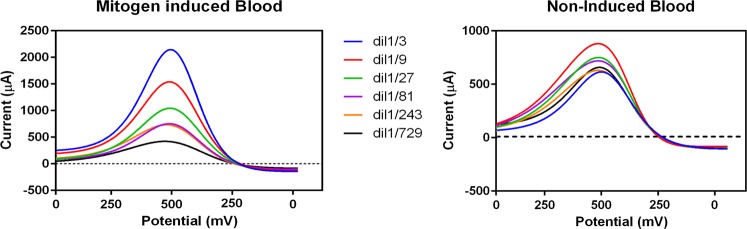
Differential voltammograms for mitogen-induced versus non-induced blood sample. Several dilutions of fresh blood in complete RPMI (RPMI-1640 medium supplemented with 10% FBS and 1% penicillin/streptomycin) used to dilute the blood samples. Wells corresponding to mitogen-induced blood received an equal amount of PHA (5 µg/mL).

The cyclic voltammogram measurement of PBMC displayed a quasi-reversible pattern. The relationship between the cell numbers and voltammograms pulse obtained in CV is shown in Fig. 2. The peak current revealed a linear relationship with cell number ranging 3 × 10^5^ to 1.2 × 10^3^ per well. The colorimetric assay (MTT OD values) also revealed a linear relationship with the same concentration of cells (number ranging 3 × 10^5^ to 1.2 × 10^3^ per well). No significant difference was observed between different concentrations of PHA. The cyclic voltammogram measurement of fresh blood also displayed a quasi-reversible pattern. The voltammograms obtained for several concentrations of mitogen-induced blood as well as non-induced blood is shown in Fig. 3.

**Figure 4:**
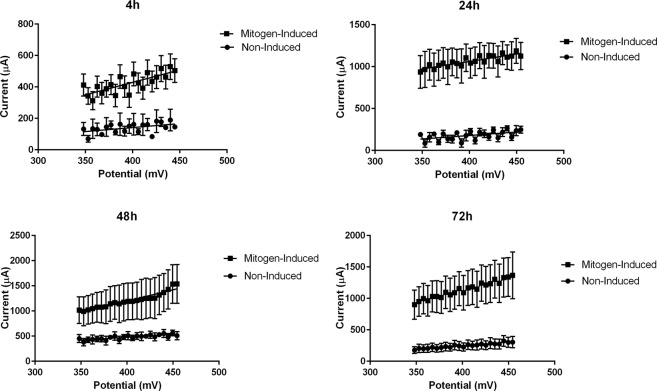
The effect of 4, 24, 48 and 72 hours incubation of cells was tested by CV (0–500 mV, 5 mV/sample). Relationship between the voltammogram pulse in the limited current values between 348 and 454 mV (23 spot) and assay duration.

The effect of 4, 24, 48 and 72 hours incubation of cells was tested by either the CV (0–500 mV, 5 mV/sample) or the optical density of the formazan produced in MTT assay. The calculated CV index (CVI), MTT OD values, and stimulation indexes for CV (CVSI) and MTT method (MTTSI) are presented in Table 2. Relationship between the voltammogram pulse in the limited current values between 348 and 454 mV (23 spot) and assay duration is shown in Fig. 4.

**Table 2:** The calculated CV index (CVI), MTT OD values, and stimulation indexes for CV (CVSI) and MTT method (MTTSI).

Assay duration	CVI		CVSI	MTT		MTTSI
	Mitogen-induced	Control		Mitogen-induced	Control	
4 h	1.62 ± 0.014	1.18 ± 0.005	1.38 ± 0.009	0.75 ± 0.05	0.69 ± 0.005	1.09 ± 0.052
24 h	1.43 ± 0.013	0.99 ± 0.015	1.44 ± 0.026	0.82 ± 0.017	0.72 ± 0.008	1.14 ± 0.021
48 h	2.95 ± 0.072	1.58 ± 0.031	1.87 ± 0.019	1.21 ± 0.02	0.91 ± 0.066	1.32 ± 0.053
72 h	2.09 ± 0.011	1.24 ± 0.003	1.69 ± 0.008	1.14 ± 0.039	0.88 ± 0.016	1.29 ± 0.047

The CVSI is expressed with average CVI value in the mitogen-induced group divided by average CVI value in negative controls. The MTTSI is expressed with average OD value in the mitogen-induced group divided by average OD value in negative controls. The PBMC cell-suspensions were adjusted at time 0 to 2 × 10^6^ cells/mL (2 × 10^5^/well) and monitoring of cell proliferation was performed at 4, 24, 48 and 72 h. Data are expressed as means ± standard error of the mean (SEM).

## Discussion

There is growing demand for developing low cost, rapid and simple analysis tools for practical applications in cellular immunology. Electrochemical techniques are an attractive candidate which provided a valuable platform for a wide range of medical applications, in particular the study of the effects of biomaterial and drugs on cell viability and toxicity^9,11^. We present here a novel application of electrochemical cell sensor for the study of lymphocyte proliferation. In this approach, cyclic voltammetry was compared with colorimetric MTT assay applicable to cell proliferation or cytotoxicity assays as an *in vitro* test.

Like other methods of proliferation assays, there are some limitations concerning the general applicability of CV method. Cyclic voltammetry is likely influenced by biologically active ingredients that may exist in different type of cells or samples (PBMC or fresh blood). Drugs or medications as well as reducing compounds are important factors to interfere with the CV assay. It has been also revealed that lymphocyte proliferation differs between individuals and in the same individual changed by the age^14^. The assay chemistry interference can be greatly reduced by using the appropriate controls and calculating the stimulation index (Treated/control ratio).

To evaluate the optimum test parameters and CVI cutoff, ROC curves were generated. ROC analysis revealed that the electrochemical responses between 0 and 500 mV, at 5 mV/sample, 100 mV/sec slope more accurately classified mitogen induced cells versus non-induced cells (Fig. 1). In order to select cut-off points, the operating characteristics of the test was adjusted for the highest sensitivity (91.6–97.6%) and specificity (97.6–100%). Accordingly, the cut-off value that maximized both sensitivity and specificity was found at the range of 348–454 mV (Fig. 4). The calculation of data derived for selected cut-off points (21 spot) by CMI formula allowed us to make an intra/inter-assay validation, as well as comparative evaluation of CV and colorimetric assays at different time points (Table 2).

To assess the electrochemical response to the cell numbers, different concentrations of cells were examined by CV assay. Each concentration showed a distinct voltammogram with minimum detectable cell number (LOD) of 1.2 × 10^3^ (Fig. 2). We also demonstrated that the same linear relationship with cell concentrations could be obtained by testing fresh blood samples. Based on the electrochemical interaction findings, one interpretation for the correlation between the cell number and current increase is the extracellular/membrane electron transfer that might be enhanced by cell intensity. Mechanisms of extracellular electron transfer and electron exchange have been well defined for bacteria and yeast^15,16^. Electroactive microorganisms can exchange electrons with other organisms or electrodes. So far, cytoplasm conductivity and membrane capacitance, as a biophysical marker, have been investigated for either white blood cells or tumor cells in suspension^17–20^. Mitogen-stimulated lymphocytes undergo alterations of their redox active products and cell surface molecules. Application of Alamar Blue (i.e. an oxidation–reduction indicator) for a lymphocyte proliferation assay, further show evidences of effectively reduction capacity in the cells undergoing proliferation^8,21^. However, the specific indirect redox mediators or direct redox cell surface-associated molecules have not been clearly identified.

In the comparison between blood induced by mitogen and non-induced blood, regarding to peak currents, two different patterns were identified (Fig. 3). A linear relationship with blood concentrations was only observed for mitogen-induced samples, diluted up to 1/81. By using CV assay a considerable significant difference was found between isolated lymphocyte and fresh blood. Our results further indicated that, although LOD differed between two experimental conditions, but voltammogram behavior drastically changed when fresh blood was induced by mitogen. According to data reported by Petty *et al*. (1995), MTT assay cannot detect less than 2.5 × 10^4^ cells per microplate well while the ATP-based assay is able to detect 1.6 × 10^3^ cells/well (i.e.) closer to CV assay^22^. The number of lymphocyte in human blood is about 2 × 10^3^ lymphocytes in 1 µL (i.e. 200 × 10^3^ per well). Intrestingly, lymphocyte number in blood at the minimum limit of detection (1/27) includes around 7 × 10^3^ cells/well that indicates the sensitivity of CV assay is still much better than the MTT method.

**Figure 5:**
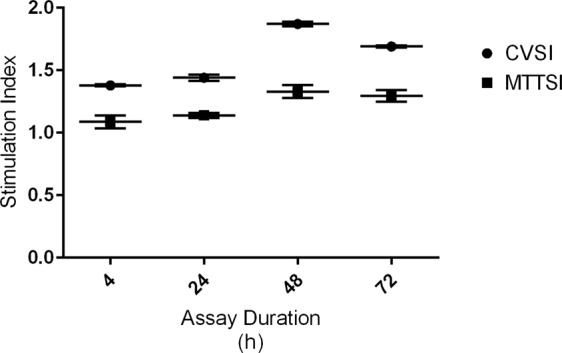
Stimulation indexes calculated for tetrazolium-based colorimetric MTT assay (MTTSI) versus stimulation indexes calculated for cyclic voltammetry (CVSI) assay during 72 hours of incubation.

The effect of cell incubation time was tested by comparing values at four time points with the same concentration of PHA (5 µg/mL) and CV parameters. In the CV assay the experimental error (SEM) slightly increased at 24 hours but remained less than MTT assay (Fig. 5). Incubation for longer time decreased the error of stimulation index. In comparison with colorimetric assay, the observed CVSI values were higher than that of MTT assay and significantly (P < 0.05) increased with time up to 48 hours after stimulation. The CV assay was able to detect the proliferative response to mitogen as early as 4 hours after induction and found to be very sensitive and rapid under the conditions examined. This will allow to analyze plates before possible chemical or physical changes during the long incubation time. Although longer incubation time will increase sensitivity, the number of cells and their metabolism can be affected by culture conditions such as altered pH, depletion of essential nutrients and accumulation of toxic by-products^23^. The eventual decline in CVSI after prolonged incubation (72 hours) might be attributed to accumulation of toxic metabolites that reduce cell proliferation. In colorimetric assay, optical density versus cell number showed a lower sensitivity to change in cell number and proliferation response. Lower test sensitivity and discrimination power was further indicated by comparing the MTTSI between different time points of incubation (Fig. 5).

Here we indicated that proliferation in cell cultures and whole blood can be monitored electrochemically using a cyclic voltammetry assay. CV assay is an efficient analytical method, since it provides a label-free, rapid, simple, high-throughput and conceivable continuous measurements. Also we could use low cost disposable electrodes to maintain the quality of results in each run. We found that this method gave the best correlation with cell count data and the sensitivity is more than colorimetric assays. *In vitro* lymphocyte proliferation assays generally require the isolation of cells, followed by a prolonged incubation time. We also demonstrated that the same clonal results could be obtained by testing fresh blood samples. In contrast, besides the advantages of short assay duration (4 hours) and rapidness, possibility use of whole blood without further processing in CV assay, would provide more accurate results because cells are monitoring in an intact environment.

## Methods

### Isolation of cells

Human blood was collected into 10 ml EDTA-anticoagulated tubes and mononuclear cells isolated by the Ficoll density gradient centrifugation method. The peripheral blood mononuclear cells (PBMC) were isolated from whole blood within 8 h of collection by centrifugation at 600 × g for 15 min through Ficoll-Hypaque Plus (Sigma–Aldrich). The white layer of cells at the plasma-Ficoll interface was harvested and washed three times with RPMI-1640 medium (Biosera, Nuaille, France) without fetal bovine serum (FBS). The cell count was measured with an automatic blood cell counter (Nihon Kohden, Tokyo, Japan). Cell viability was determined with a hemacytometer, using trypan blue exclusion method. Samples with the number of viable cells less than 95% and red blood cell contamination more than 2% were excluded from further tests.

This study was approved and monitored by National Research Ethics Committee, University of Tehran. All methods were performed in accordance with the relevant guidelines and regulations of the institution. Informed consent was obtained from subjects.

### Lymphocyte proliferation studies

Duplicate wells in each plates were set-up for concurrent determination of proliferation by colorimetric MTT (tetrazolium) and cyclic voltammetry (CV) assays. One hundred microliters of diluted cell suspension (2 × 10^6^ cells/mL) in complete RPMI (RPMI-1640 medium supplemented with 10% FBS and 1% penicillin/streptomycin) were dispersed and incubated in 96-well microtiter plate. Cells were seeded in 10 wells per sample for each test (mitogen added) and control negative experiment. The mitogen phytohemagglutinin-L (PHA-L; Sigma) at the final working concentrations of 5, 10 and 20 µg/mL was added to test wells. In addition, cell-free media containing complete RPMI were run in parallel to test and control groups. The inoculated plates were incubated at 37 °C in a 5% CO_2_ incubator and checked for growth after 4, 24, 48 and 72 h using both colorimetric and CV assays. Each assay is further evaluated for sensitivity to change in cell number and different concentrations of mitogen. Six different concentrations of cells in complete RPMI media ranging from 3 × 10^6^ to 1.2 × 10^4^ cells/mL (3 × 10^5^ − 1.2 × 10^3^ cells/well) were tested by both colorimetric MTT and CV assays. All the experiments reported here were repeated four times.

### Colorimetric MTT assay

Colorimetric lymphocyte proliferation was evaluated using MTT (3-(4, 5-dimethyl thiazol-2-yl) 2, 5-diphenyl tetrazolium bromide) method. After cell culture incubation (4, 24, 48 and 72 h), 10 μl MTT (dissolved in RPMI-1640, 5 mg/mL) was added to well, and plates were further incubated at 37 °C for 4 h. Finally, the purple formazan crystals were dissolved by adding 100 μl acid-isopropanol (0.04 N HCI in isopropanol) into each well. Absorbance was then measured at 550 nm against reference wavelength of 630 nm and stimulation index (SI) was determined. The SI is expressed with average OD value in the test group divided by average OD value in negative controls.

### Cyclic voltammetry (CV) assays

Cyclic voltammogram measurement was performed using a home-made potentiostat which was designed according to the needs. One of which was to be able to measure currents up to 10 micro amps, and the other was to be able to change slope, voltage boundaries applied to the sample and also the sampling rate. A mini-electrochemical system containing three-electrode system was constructed according to the previously described method^24,25^. A polished pencil graphite was used as the working electrode, a platinum wire as the auxiliary electrode and a saturated Ag/AgCl electrode as the reference electrode. The voltage applied to the samples was set in a way to prevent cells from dying due to oxidation. So the cells kept alive along sampling time and after. To gain more information on the voltammetric behaviors of cells, current-voltage (I-V) spectra were obtained at four different potential sets including 0–250 V, 0–500 mV, 0–750 mV and 0–1000 mV with the same sweep rate of 5 mV/sample at the slope of 100 mV/sec. Voltammograms were obtained at room temperature (22 ± 2 °C) and data analyzed to find the best voltage ranges and other specifications.

### Testing fresh blood by CV assay

Two hundred microliter of collected blood sample containing 1.8 × 10^6^ lymphocyte/mL (1.8 × 10^5^/well) was placed in 96-well sterile culture plates and serially diluted three-fold with complete RPMI. The PHA at the final working concentrations of 5 µg/mL was added to test wells. As a negative control, the same serially diluted blood in RPMI media without PHA were run in parallel to test group. The plate was incubated at 37 °C in a 5% CO_2_ incubator and test by CV after 4 h.

### Data analysis

Since the using CV for the lymphocyte proliferation assay represents a novel format, for all the possible and appropriate cut-off values of the test, receiver-operator characteristic (ROC) curves are constructed. Accordingly, for each point of CV data the sensitivity was plotted against one minus specificity for all the possible cut-off values. The values that provides an operating position nearest that of 100% sensitivity and 100% specificity were selected based on Youden’s index (J). The Youden’s J statistic, which shows the performance of a diagnostic test, was calculated as sensitivity + specificity −1; maximum value of the index was used as a criterion for selecting the optimum cut-off point.

Between-group comparisons were performed by one-way analysis of variance (ANOVA) using Fisher’s protected least significant difference set to the 95% confidence level. Correlations were assessed by linear regression analysis. Repeated measure analysis was conducted to test the PHA treatment effect on the cell proliferation data during the different time point of experiment. Results are expressed as mean ± S.E.M. of absolute values. All statistical analyses were carried out with the software SPSS ver. 23.0.0.1 (Chicago, IL, USA).

## Data Availability

The datasets generated and/or analysed during the current study are not publicly available due [because it is not general data] but are available from the corresponding author on reasonable request.
